# Editorial: Investigation of the biological properties of natural products using experimental approaches and *in silico* methods

**DOI:** 10.3389/fchem.2024.1406883

**Published:** 2024-04-17

**Authors:** Suraj N. Mali, Cleydson Breno Rodrigues dos Santos, Joaquín Campos Rosa, Jorddy Neves Cruz

**Affiliations:** ^1^ School of Pharmacy, D.Y. Patil University (Deemed to be University), Navi Mumbai, India; ^2^ Laboratory of Modeling and Computational Chemistry, Department of Biological and Health Sciences, Federal University of Amapá, Macapa, Brazil; ^3^ Department of Pharmaceutical and Organic Chemistry, Faculty of Pharmacy, Campus of Cartuja, University of Granada, Granada, Spain; ^4^ Institute of Biological Sciences, Federal University of Pará, Belém, Brazil

**Keywords:** natural compounds, biological properties, computer aided drug design (CADD), extraction of compounds, medicinal chemistry and drug synthesis

Natural products sourced from plants, animals, and microorganisms, offer diverse biological activities that are crucial for drug discovery (Rout et al., 2022). Traditionally, experimental methods like biological assays and spectroscopic analyses have been used alongside compound synthesis to explore their properties. However, computational techniques such as molecular docking and machine learning are now complementing these approaches, facilitating the investigation of natural product interactions with biomolecules. Integrating experimental and computational methods accelerates the discovery of bioactive compounds, the optimization of efficacy, and the elucidation of mechanisms. For example, computational screening identifies potential candidates from large natural product databases, which are then validated by experimental assays. This fusion of experimental and computational strategies is revolutionizing drug discovery, promising novel therapeutics for various diseases.

Additionally, *Phanera splendens* (Kunth) Vaz., a medicinal plant used in traditional medicine against malaria, harbors highly efficient endophytic bacterial isolates with biocontrol properties. *Bacillus* sp. produces non-ribosomally synthesized cyclic lipopeptides, notably surfactins, which exhibit antimicrobial activity, including against *Plasmodium falciparum*. Scientific evidence suggests that surfactin structure 2d-01 is a potent inhibitor of *P. falciparum sirtuin (Sir2)* (PfSir2A), a regulator of *P. falciparum* growth and gene expression (Nagtode et al., 2023). The study reported by de Souza et al. showed that six surfactins were produced by endophytic bacteria, with *in silico* analysis revealing the binding mode of surfactins at the active site of the PfSir2A enzyme. Among them, 1d-02 exhibited the highest affinity, suggesting its potential as an antimalarial compound. This insight into surfactin structures and their interaction with the PfSir2A enzyme could aid in the design of new antimalarial compounds (de Souza et al.). Flores-Holguín et al. characterized four marine toxins, two hydrophilic and two lipophilic compounds, and assessed their drug-like properties and reactivity in different geometries. Domoic Acid (DA) exhibited strong target interaction and stability with low hardness and potential surface area, meeting the Rule of Five parameters (Flores-Holguín et al.). It demonstrated high gastrointestinal (GI) absorption and optimal properties in terms of lipophilicity, molecular weight, polarity, solubility, saturation, and flexibility. In another study, Lone et al. discovered two steroidal saponins, trilliumosides K (1) and L (2), from *Trillium govanianum* rhizomes in addition to seven known compounds. Spectroscopic analysis, including 1D and 2D NMR and HR-ESI-MS, confirmed the structures. Compound **1** showed significant cytotoxic activity against A-549 and SW-620 cancer cell lines (IC_50_ values: 1.83 µM and 1.85 µM, respectively), while Compound **2** had an IC_50_ of 1.79 µM against A-549 cells. Known compounds 3, 5, and 9 exhibited IC_50_ values between 5 and 10 µM. Compound 2 also inhibited migration and colony-forming capability in A-549 cells, inducing apoptosis by altering nuclear morphology, increasing ROS production, decreasing MMP levels, and modulating BAX and BCL-2 proteins, and activating Caspase-3 (Lone et al.).

Gallo et al. discovered that an organic extract from the Mediterranean ascidian *Ciona robusta* inhibited cell proliferation in HT-29, HepG2, and U2OS human cells, with HT-29 being the most sensitive (EC_50_ = 250 μg/mL). The extract did not affect the viability of HT-29 cells induced to differentiate with sodium butyrate, indicating a preference for the malignant phenotype. Cell death induced by the extract was attributed to cytotoxic autophagy, as evidenced by increased LC3-II expression and autophagic vacuole formation in HT-29 cells. Although the exact composition of the extract remains unknown, it contains bioactive compounds with anticancer properties. In another study by Rocha et al., a computational approach was used to investigate potential antivirals against SARS-CoV-2 Mpro. The authors screened 288 flavonoids from Brazilian biodiversity, selecting 204 compounds based on Lipinski’s rule of five (Rocha et al.). These compounds were docked into Mpro’s active site and re-scored using MM-GBSA binding free energy calculations. The top five flavonoids were analyzed for their interactions with Mpro residues. Additionally, another report focused on the anti-leishmanial activity of *Eleutherine plicata* Herb. and predictions regarding isoeleutherin and its analogs (Albuquerque et al.).

Mutran et al. investigated essential oil-containing mouthwashes for dental enamel preservation, aiming to release low concentrations of Ca and P without altering morphology (Mutran et al.). This study highlights the potential of natural essential oils for health applications. Yin et al. explored the molecular mechanisms of *Uncaria rhynchophylla*-*Alisma plantago-aquatica* L. (UR-AP), a traditional remedy for hypertension, using network pharmacology, cluster analysis, and molecular docking (Yin et al.). They identified 58 bioactive compounds, including quercetin and beta-sitosterol, and 143 common targets between UR-AP and hypertension. Key targets such as MAPK1 and IL6 were linked to pathways critical for the treatment of hypertension. Molecular docking validated the interactions, suggesting UR-AP as a novel hypertension therapy. While promising, *in vivo* validation is required. In another study by Lata et al., two proanthocyanidins were isolated from *Ficus glomerata* and assessed for bioactivity using OSIRIS property explorer applications (Lata et al.). Predictive drug scores indicated potential biological effects, and *in vitro* testing against ruminal enzymes showed significant inhibitory activities, suggesting that these compounds may impact rumen enzyme biology and hold promise for drug development.
